# Barusiban suppresses oxytocin-induced preterm labour in non-human primates

**DOI:** 10.1186/1471-2393-7-S1-S15

**Published:** 2007-06-01

**Authors:** Torsten M Reinheimer

**Affiliations:** 1 Non-Clinical Development, International PharmaScience Center, Ferring Pharmaceuticals A/S, Kay Fiskers Plads 11, 2300 Copenhagen S, Denmark

## Abstract

**Background:**

Preterm labour (PTL) is a major cause of neonatal mortality and morbidity, and oxytocin (OT) antagonists are potential tocolytics. Atosiban (TRACTOCILE) is a mixed vasopressin V_1A_/OT antagonist registered for acute treatment of PTL in Europe. Other off-label drugs have serious side effects. Barusiban is a selective OT antagonist which has reached clinical development. A monkey model with OT-induced PTL was developed to compare barusiban and atosiban. In addition, the feasibility for long-term treatment of PTL with barusiban was explored.

**Methods:**

Conscious pregnant cynomolgus monkeys were monitored for intrauterine pressure (IUP). A sensor for IUP was implanted into the amniotic cavity, and biopotential sensors for electromyogram were attached to the uterus. For short-term experiments, individual low-dose OT infusions induced stable submaximal uterine contractions. Barusiban and atosiban were administered either as intravenous bolus or infusion at high or low doses. For long-term treatment, low-dose OT was infused daily for 3–6 hours to mimic PTL. In addition, continuous high-dose infusions of barusiban (150 μg kg^-1 ^h^-1^) or fenoterol (3 μg kg^-1 ^h^-1^) were administered.

**Results:**

Contractions of 15–40 mmHg were induced with individual OT infusions at 5–90 mU kg^-1 ^h^-1^, and no OT-related desensitization occurred. Correlation was demonstrated between electromyograms and IUP curves. Barusiban was well tolerated and its potency was 4 times higher than atosiban's. Barusiban and atosiban demonstrated >95% efficacy. However, barusiban's duration of action was >13 hours (atosiban's 1–3 hours) and reversible with high-dose OT in emergency situations. OT control and fenoterol-treated monkeys delivered preterm (*ca*. day 154) and showed an increase in overall IUP. Barusiban-treated animals delivered normally following end of treatment (*ca*. day 163).

**Conclusion:**

The presented telemetry model provides an excellent method to evaluate PTL drug candidates. OT induced stable repetitive contractions and no desensitisation. Barusiban and atosiban demonstrated high efficacy and rapid onset of action. Barusiban, a selective OT antagonist has higher potency and prolonged duration of action than atosiban. Barusiban effectively suppressed IUP during daily OT-challenges, delayed labour, and prolonged monkeys' pregnancy till term.

## Background

PTL is associated with neonatal morbidity and mortality and represents an unmet clinical need. The incidence ranges from 5–25 percent, shows an increasing trend, and has a significant economic and social impact [1-7]. Many preterm infants suffer from health problems, such as neurosensory deficits, cerebral palsy, respiratory distress, gastrointestinal problems, growth deficits, and mental retardation [8]. Endocrine OT from the hypothalamic-pituitary axis and paracrine OT from the uterine/fetal compartment are thought to drive labour and parturition through its receptors [9-11]. Thus, OT antagonists are potentially useful therapeutic agents to delay PTL and delivery.

Atosiban (TRACTOCILE) is a mixed vasopressin V_1A _(preferentially) and OT receptor antagonist [12]. It is the first tocolytic specifically developed for management of PTL and approved in Europe and many other countries [13]. Atosiban can be used for short-term treatment (typically 48 hours) to delay imminent preterm birth between 24 and 33 weeks of gestation. This provides the chance to reduce respiratory distress syndrome by administration of antenatal glucocorticoids and allows time for transfer *in utero *to a neonatal intensive care unit. Most other tocolytics are used off-label, compromised by side effects for mother and child, or offering limited efficacy.

Barusiban is a new long-acting OT receptor antagonist developed for PTL. It is a cyclic heptapeptide and an analogue of endogenous OT designed for longer duration of action [12]. This could provide the convenience of less frequent administration in the clinic. Barusiban has approximately 300-fold greater affinity for the human cloned OT receptor than for the V_1A _receptor, whereas atosiban binds well to both receptors. In contractility studies with isolated human myometrium, barusiban also demonstrates more OT selective inhibitory effects [14]. It has reached phase II of clinical development as a tocolytic.

Here, a non-human primate model for PTL is presented that was developed to ensure full GLP compliance. Radiotelemetry was employed to measure electromyograms (EMG) and IUP, while remote dosing and blood sampling was established. OT was used to induce stable uterine contractions, and respective desensitisation was examined. A detailed pharmacokinetic/pharmacodynamic (PK/PD) comparison of barusiban with atosiban was performed in terms of onset and duration of tocolysis, efficacy, and potency. A rescue treatment was included to verify the reversibility of tocolysis with OT. Finally, the feasibility of long-term treatment with barusiban was demonstrated, including a comparison with fenoterol, a *β*_2 _adrenergic receptor agonist.

## Methods

**Figure 1:**
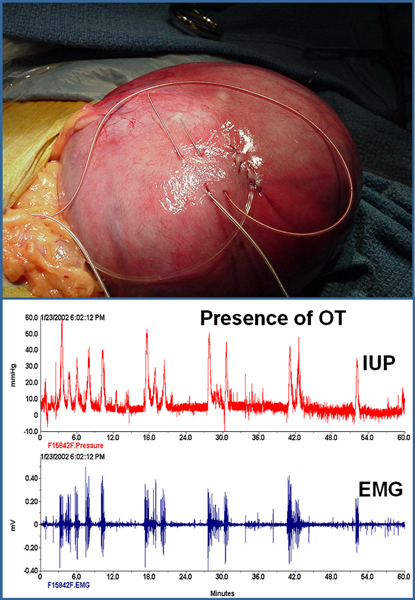
**Instrumented uterus and corresponding EMG and IUP correlation**. **Top: **Chronically instrumented uterus of a pregnant cynomolgus monkey *in situ *at GD 120, showing two bio-potential electrodes attached to the myometrium for measurement of electromyograms (EMG, scale: electrodes are 1 cm apart) [15]. A tube was inserted into the amniotic cavity for registration of the respective intrauterine pressure (IUP). **Bottom: **Correlation between uterine EMG (blue) and IUP (red). Typical bursts and corresponding contractions occur every 3–6 min. The highest spontaneous activity was observed in the night close to birth. Note the close relationship between bursts of electrical activity in the EMG and increases in IUP, here in the presence of OT infusion.

**Figure 2:**
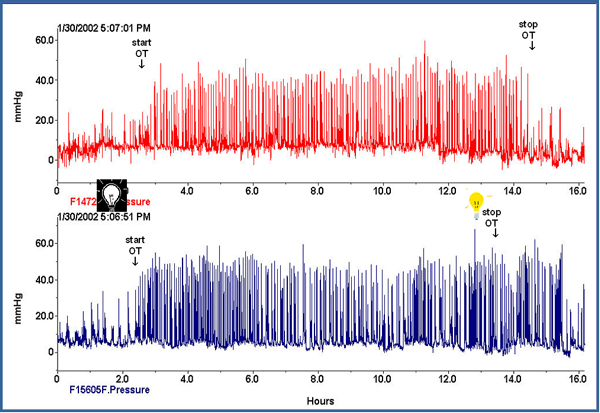
**Oxytocin-induced uterine contractions in cynomolgus monkeys**. Individual infusion of 5–90 mU OT kg^-1 ^h^-1 ^induced stable uterine contractions of about 15–40 mmHg [15]. No short- or long-term desensitisation of OT-induced contractions was observed. On the background of these stable OT-induced contractions, different tocolytics have been evaluated in terms of their PK/PD characteristics. Here two monkeys were infused in parallel for about 11 hours during the night. The switch-off the lights in the evening (06:00 PM) and switch-on the lights (06:00 AM) is labelled by respective symbols.

A telemetric-based model is presented for evaluation of uterine contractions in pregnant cynomolgus monkeys [15]. A pressure sensor was implanted into the amniotic cavity and biopotential sensors were attached to the uterus on gestational day (GD) 120 ± 3 (Figure 1). A telemetry transmitter was placed in a subcuticular pocket located in the flank. The model allows continuous monitoring of EMG and IUP as indicators of uterine activity. Venous catheters were connected to the next room for remote dosing and blood sampling for plasma analysis, without disturbing the conscious animals or causing iatrogenic influences on uterine contractions. OT was individually infused at 5–90 mU kg^-1 ^h^-1 ^to induce artificial contractions of 15–40 mmHg that mimicked PTL (Figure 2). All protocols and procedures were approved by the local Animal Care and Use Committee and according to guidelines for laboratory animals.

During short-term treatment a total eight monkeys were included, and each group consisted of three (or two) pregnant females [16]. The groups participated in up to four phases of treatment that were separated by a 24–48 h washout period. The first treatment phase generally started on GD 128. OT control infusions were given at the lowest dose required for stable and submaximal contractions in each individual female. The treatment phases included: OT control, OT + barusiban, OT + atosiban, and OT + barusiban and then escalating high-dose OT rescue therapy (133–2000 mU kg^-1^ h^-1^). Dose levels of barusiban and atosiban were 10–50 and 100–500 μg kg^-1 ^(bolus) and 2.5–150 and 50–250 μg kg^-1 ^h^-1 ^(infusion for 2–3 h), respectively. These dose levels were selected to be in the range of half-maximal to maximal efficacy, based on pre-existing PK/PD data.

In long-term treatment, each group consisted of three (or two) pregnant females [17]. The pregnant females were dosed from *ca*. GD 141 to 163 or delivery, whichever came first. OT was intravenously (IV) infused for 3–6 h per day to simulate a situation of PTL with daily periods of spontaneous contractions. Barusiban, fenoterol, or saline (control animals) were administered by continuous IV infusion for 24 h per day. The dose level for barusiban (150 μg kg^-1 ^h^-1^) was determined according to maximal effects observed previously [16]. Atosiban was not further investigated due to the limited number of animals. Fenoterol is approved for treatment of PTL and used in this study as a comparative control. The dose level for fenoterol (3 μg kg^-1 ^h^-1^) was based on the maximally effective dose cited in the package insert, adjusted for the body weight of monkeys.

## Results

### Method validation

The animals underwent surgery at about GD 120, and the instrumentation for EMG, IUP, and dosing/blood sampling was well tolerated; the monkeys recovered within one week [15]. There was a good correlation between the electrical bursts in the EMG and the increase in IUP (Figure 1). Typically contractions occurred every 3–6 min. The highest spontaneous activity was observed in the first half of the night with increasing intensity close to birth. Stable contractions for comparison of the tocoloytics were induced by individual infusion of 5–90 mU kg^-1 ^h^-1 ^OT during the nights (Figure 2). No desensitisation of OT-induced contractions was observed.

### Short-term treatment

**Figure 3:**
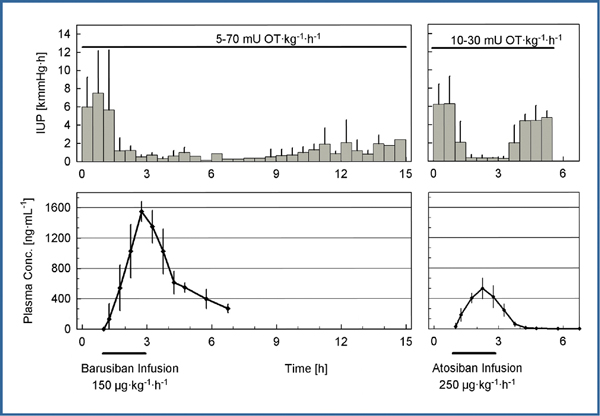
**Barusiban and atosiban effects on OT-induced contractions**. **Top left: **A two hour high-dose IV infusion of barusiban at 150 μg kg^-1 ^h^-1 ^decreased IUP within 0.5 h, compared with OT control levels, and the effect was sustained for more than 14 h [16]. Barusiban has a longer duration of action when compared with atosiban. IUP curves were integrated as area under the curve in half hour intervals. Data shown are means ± SD for three animals (n = 1–2 when SD not shown). **Top right: **A two-hour high-dose IV infusion of atosiban at 250 μg kg^-1 ^h^-1 ^produced similar reductions in IUP, but the effect lasted for only 3-h duration. Both antagonists demonstrated an immediate onset of inhibition of uterine contractions and a high efficacy. **Bottom left: **PK data showed that the effects of barusiban persisted during declined plasma levels. The plasma samples were analysed with liquid chromatography and tandem mass-spectroscopy. **Bottom right: **Atosiban effects occurred only during periods of peak plasma concentrations.

Following induction of stable contractions by OT, barusiban or atosiban were administered as high- or low-dose bolus or infusion [16]. Both antagonists showed an immediate onset of action and full inhibitory efficacy (Figure 3). However, barusiban's duration of action was generally longer than 13–15 hours, while atosiban's effect ceased within 1.5–3 hours (Table 1). The calculated potency of barusiban was about four times higher than the potency of atosiban.

In rescue treatments, the reversibility of barusiban's inhibition of contractions was demonstrated. First, stable contractions were established by low-dose OT infusion and then a high-dose bolus of barusiban lead to full inhibition of contractions. Finally, escalating high-dose OT infusion reinstated the uterine contractions.

During the experiments a good EMG IUP correlation could be shown. Following barusiban treatment and a respective decrease in IUP, there was in parallel a decreased electrical burst duration, an increased number of bursts, and a decrease in the power density spectrum (PDS) peak frequency (data not shown).

### Long-term treatment

**Figure 4:**
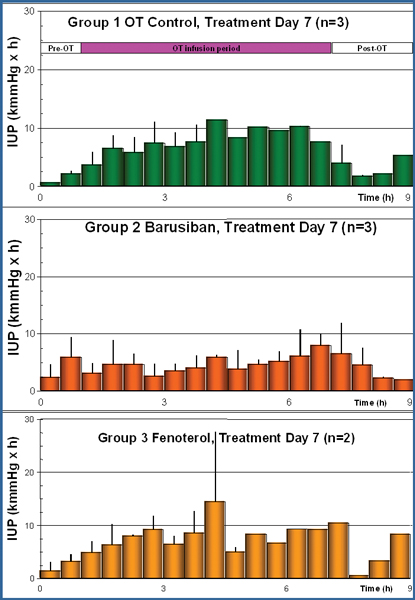
**Daily comparison during long-term treatment**. Representative IUP values from treatment day 7 in pregnant monkeys continuously IV infused with saline (control in green), 150 μg kg^-1 ^h^-1 ^barusiban (in orange), or 3μg kg^-1 ^h^-1 ^fenoterol (in yellow) [17]. All groups were challenged daily with OT infusion for 3–6 hours (represented by the pink bar). Barusiban was effective in suppressing OT-induced increases in uterine contractility, whereas fenoterol was not. Barusiban was statistically superior to the OT control on 8/17 days and better than fenoterol on 11/17 days. Data shown are means ± SEM. Intervals without SEM are data for individual animals, which occurred because of varying duration of OT infusion or limited post-OT measurements. The unit of the integrated half-hour IUP intervals [kmmHg h] is kilo millimetre mercury times hour.

The animals were continuously infused with saline control, barusiban, or fenoterol [17]. During daily 3–6 hours OT infusion, the control and fenoterol groups showed an increase in IUP that was reduced in barusiban-treated animals (Figure 4). In 8/17 days and 11/17 days, the barusiban group had statistically lower IUP than the control and fenoterol groups, respectively.

**Figure 5:**
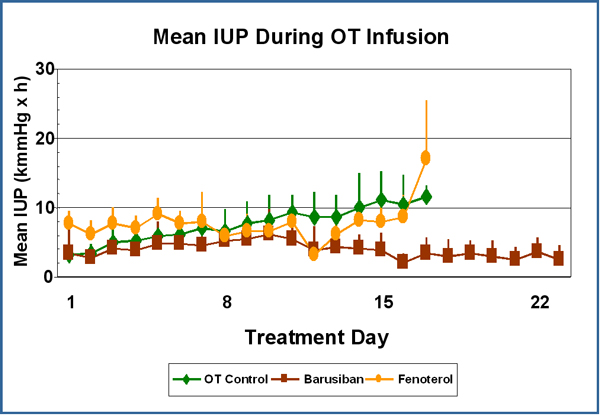
**Intrauterine pressure progression during long-term treatment**. Barusiban inhibited OT-induced increases in IUP in near-term pregnant monkeys (GD *ca*. 141 to 163 or delivery) [17]. In general, IUP was lowest throughout the last about three weeks of pregnancy in barusiban-treated females (150 μg kg^-1 ^h^-1^, orange), compared to OT controls (green), or fenoterol-treated females (3 μg kg^-1 ^h^-1^, yellow). Barusiban was particularly effective in maintaining low IUP near the end of gestation. OT control and fenoterol-treated animals gave preterm birth, barusiban-treated animals continued pregnancy till term, when the treatment was stopped, and the mothers delivered normally. Data shown are means ± SEM for n = 3 (controls, barusiban) or n = 2 (fenoterol).

During long-term IUP progression, barusiban was effective in maintaining low IUP during daily OT-challenges (Figure 5). OT controls and the fenoterol group showed a marked IUP increase 4–5 days prior to delivery which occurred generally after 17 days of treatment. The barusiban group continued pregnancy until the end of approximately 23 treatment days and delivered afterwards.

The basal overall IUP before the daily OT challenges was unchanged during barusiban or fenoterol treatment compared to control. During OT infusion, about a fourfold increase occurred in overall IUP. In fenoterol-treated animals, the overall IUP increased approximately threefold. However, in the barusiban group, there was only a 30 percent rise in overall IUP.

## Discussion

The availability of a telemetric uterine contraction model in preterm cynomolgus monkeys is an excellent reproductive tool for evaluation of tocolytic drug candidates [15]. The model does not interfere with maternal/fetal health and is not impacted by iatrogenic disturbances or stress. The surgery and instrumentation was well tolerated and allows evaluation of uterine activity and PK/PD relationships in response to exogenously administered drug candidates. OT was used as a means to induce stable PTL-like contractions. Following OT infusion, neither short-term nor long-term desensitisation occurred. Because of the good EMG/IUP correlation, EMG may be an interesting clinical tool for evaluation of uterine contractions in the future.

The OT system is known to play a key role in the initiation and maintenance of labour, including PTL [17]. In consequence, OT antagonists are logical candidates for pharmacological management of PTL. The two OT receptor antagonists, barusiban and atosiban, both have high efficacy and rapid onset of action when evaluated for inhibition of OT-induced uterine contractions [16]. Furthermore, the inhibitory effects of barusiban were reversible by administration of high-dose OT. This confers control over the duration of inhibition, especially in the case of emergency delivery by caesarean section. Barusiban has higher potency and longer duration of action than atosiban.

Barusiban is both safe and efficacious in reducing uterine activity in response to daily OT challenge in a non-human primate model that mimics long-term treatment in PTL. When administered by continuous infusion for the last *ca*. 3 weeks of pregnancy, barusiban prevented early birth induced by OT and extended pregnancy to normal duration. Since neonatal survival rates improve three percent with each day that pregnancy can be extended [18], this might convey substantial socioeconomic benefit. This is the first proof of principle that barusiban is effective for long-term management of PTL-like contractions. Now, clinical trials have to verify barusiban's effectiveness compared to its effect on OT-induced PTL in non-human primates.

## Conclusion

Taken together, the presented model is useful for PK/PD evaluation of drug candidates intended to be used in PTL. Continuous low-dose OT infusions induce stable uterine contractions. A good correlation between EMG and IUP was observed.

Barusiban suppressed OT-induced PTL-like uterine contractions with immediate onset of action and high efficacy. Compared with atosiban, it had a higher potency and longer duration of action. Barusiban's inhibition could be reversed by high-dose OT.

During long-term treatment, barusiban reduced IUP in OT-challenged monkeys; it delayed the onset of labour and lead to a prolonged pregnancy. Barusiban is suggested as a safe and effective tocolytic for acute and maintenance treatment.

## Competing interests

The author is employed by Ferring Pharmaceuticals A/S.

## Authors' contributions

TMR was the sponsor's monitor and proposed the study design. He was involved in protocol development and data interpretation, performed study audits, and report reviews. He drafted the manuscript and presented it at the PTL meeting in Tarragona.

## Figures and Tables

**Table 1:** Summary of barusiban and atosiban PK/PD characteristics

**Pharmacodynamics (PD)**	**Barusiban**	**Atosiban**
Onset of action, hours	≤0.5–1.5	≤0.5–1.0
Efficacy (decrease in IUP), %	≤98	≤96
Duration of action, hours	> 13–15	1–3
Potency (IC_50_), ng/ml	12.7	47.4

**Pharmacokinetics (PK)**	**Barusiban**	**Atosiban**

Half-life, hours	1.46–2.57	0.46–0.66
Clearance, ml h^-1 ^kg^-1^	25–66	539–936
Distribution, ml kg^-1^	70–159	359–640

The values in the table represent the overall results obtained for barusiban and atosiban given IV as high- or low-dose bolus or infusion, respectively to pregnant cynomolgus monkeys [16]. The plasma samples were analysed with liquid chromatography and tandem mass-spectroscopy.
